# Isolation of *Listeria ivanovii* from Bulk-Tank Milk of Sheep and Goat Farms—From Clinical Work to Bioinformatics Studies: Prevalence, Association with Milk Quality, Antibiotic Susceptibility, Predictors, Whole Genome Sequence and Phylogenetic Relationships

**DOI:** 10.3390/biology11060871

**Published:** 2022-06-06

**Authors:** Daphne T. Lianou, Anargyros Skoulakis, Charalambia K. Michael, Eleni I. Katsarou, Dimitris C. Chatzopoulos, Nikolaos Solomakos, Katerina Tsilipounidaki, Zoe Florou, Peter J. Cripps, Angeliki I. Katsafadou, Natalia G. C. Vasileiou, Konstantina S. Dimoveli, Maria V. Bourganou, Dimitra V. Liagka, Vasileios G. Papatsiros, Panagiota I. Kontou, Vasia S. Mavrogianni, Mariangela Caroprese, Efthymia Petinaki, George C. Fthenakis

**Affiliations:** 1 Veterinary Faculty, University of Thessaly, 43100 Karditsa, Greece; dlianou@vet.uth.gr (D.T.L.); cmichail@vet.uth.gr (C.K.M.); elekatsarou@vet.uth.gr (E.I.K.); nsolom@vet.uth.gr (N.S.); peterjohncripps@gmail.com (P.J.C.); kdimoveli@uth.gr (K.S.D.); vpapatsiros@vet.uth.gr (V.G.P.); vmavrog@vet.uth.gr (V.S.M.); 2 Institute for Bioinnovation, BSRC Alexander Fleming, 16672 Vari, Greece; skulakis@gmail.com; 3 Faculty of Public and One Health, University of Thessaly, 43100 Karditsa, Greece; vetdchatzop@gmail.com (D.C.C.); agkatsaf@vet.uth.gr (A.I.K.); mbourganou@uth.gr (M.V.B.); 4 University Hospital of Larissa, 41110 Larissa, Greece; tsilipoukat@gmail.com (K.T.); zoi_fl@yahoo.gr (Z.F.); petinaki@med.uth.gr (E.P.); 5 Faculty of Animal Science, University of Thessaly, 41110 Larissa, Greece; vasileiounat@gmail.com (N.G.C.V.); dliagka@vet.uth.gr (D.V.L.); 6 Department of Mathematics, University of Thessaly, 35131 Lamia, Greece; pankontou@gmail.com; 7 Department of Agriculture, Food, Natural Resources and Engineering (DAFNE), University of Foggia, 71122 Foggia, Italy; mariangela.caroprese@unifg.it

**Keywords:** goat, Greece, *Listeria*, milk, phylogenetic analysis, sheep, virulence factors, whole genome sequence

## Abstract

**Simple Summary:**

An extensive countrywide study in Greece revealed that isolation of the zoonotic pathogens *Listeria* *monocytogenes* and *Listeria ivanovii* from the milk produced in sheep or goat farms was infrequent: 1.2% of farms sampled. The presence of pigs on the farms, low average relative humidity in the environment and a high number of animals on the farms were found to be associated with the isolations. Detailed assessment of the *L. ivanovii* strains, for which there is a paucity of information worldwide, revealed that the isolated strains belonged to the *L. ivanovii* subsp. *ivanovii* branch. All strains of the branch appeared to be very similar, with the distance between them being small, which suggests that global spread of this clonal branch is a recent evolutionary event or that the branch is characterized by a slow evolutionary rate.

**Abstract:**

A cross-sectional study was performed in 325 sheep and 119 goat dairy farms in Greece. Samples of bulk-tank milk were examined by standard microbiological techniques for *Listeria* spp. *Listeria monocytogenes* was isolated from one (0.3%) and *Listeria ivanovii* from three (0.9%) sheep farms. No associations between the isolation of *L. monocytogenes* or *L. ivanovii* and milk quality were found. No resistance to antibiotics was identified. Three variables emerged as significant predictors of isolation of the organism: the presence of pigs, low average relative humidity and a high number of ewes on the farm. The three *L. ivanovii* isolates were assessed in silico for identification of plasmids, prophages, antibiotic resistance genes, virulence factors, CRISPRs and CAS genes. Phylogenetic analysis using the core genome revealed that the three strains belonged to the *L. ivanovii* subsp. *ivanovii* branch and were especially close to the PAM 55 strain. All strains of the branch appeared to be very similar, with the distance between them being small.

## 1. Introduction

*Listeria ivanovii* belongs to the family Listeriaceae and was first identified in 1984 [1]. The species includes two subspecies, namely *L. ivanovii* subsp. *ivanovii* and *L. ivanovii* subsp. *londoniensis* [2]. *L. ivanovii* is one of the two pathogenic *Listeria* species (the other being *Listeria monocytogenes*) and is included in the *Listeria* sensu stricto clade [3]. The whole genome sequence of *L. ivanovii* subsp. *ivanovii* was first reported in 2011 and referred to an isolate from an outbreak of abortion in sheep in Spain (reference strain: PAM 55; serotype 5) [4].

The relevant international literature on the organism is limited. A search in the Web of Science database under the term ‘*Listeria ivanovii*’ and the subsequent detailed evaluation of the returned records revealed a total of 355 relevant original articles that have been published up to the end of 2021 (Table S1). The organism is an infrequent but confirmed pathogen of ruminants. There are a few reports of isolation of the organism from clinical samples from sheep [5,6], including milk samples [7,8], as well as from goats [9,10], cattle [11,12] and buffaloes [13] In ruminants, the organism has been mainly associated with abortion [14]. Isolation of the organism from other animal species has rarely been reported. There are also some reports of isolation of the organism from clinical samples collected from humans (United Kingdom (*n* = 2), France (*n* = 2), Germany, Israel and Spain), mainly from immunocompromised patients [15,16]. Human infections are mainly of foodborne origin [17], which indicates a need for an evaluation of the presence of the pathogen in ruminants and their products, whence it may spread to animal products.

Greece has a high number of sheep and goats, around 8,400,000 and 3,600,000, respectively [18], which account for approximately 6.5% and 22.0%, respectively, of the total number of small ruminants in Europe [19]. The respective milk production from these animals amounts to 645,000 and 350,000 tons annually [19,20]. Milk from sheep and goats is of particular significance for the Greek agricultural sector because of its increased use in dairy products.

This study presents the results of an extensive countrywide investigation performed in 325 dairy sheep flocks and 119 goat herds throughout Greece. The objectives of the study were (a) to investigate the possible presence of *Listeria* spp. in the bulk-tank raw milk of small-ruminant farms in Greece; (b) to identify the factors potentially associated with the presence of the bacteria in bulk-tank raw milk; (c) to assess the antibiotic susceptibility patterns of the isolates obtained; (d) to study the whole genome sequence of the isolates obtained, with special reference to *L. ivanovii* strains, and define antibiotic resistance genes, virulence factors genes, mobile elements and CRISPR/CAS locus (clustered regularly interspaced short palindromic repeats/CRISPR-associated genes) present on the genome and (e) to reveal the phylogenetic relationships of the genomes of the strains of *L. ivanovii* obtained during the study in comparison with strains previously isolated.

## 2. Materials and Methods

### 2.1. Farms and Sampling

**Figure 1:**
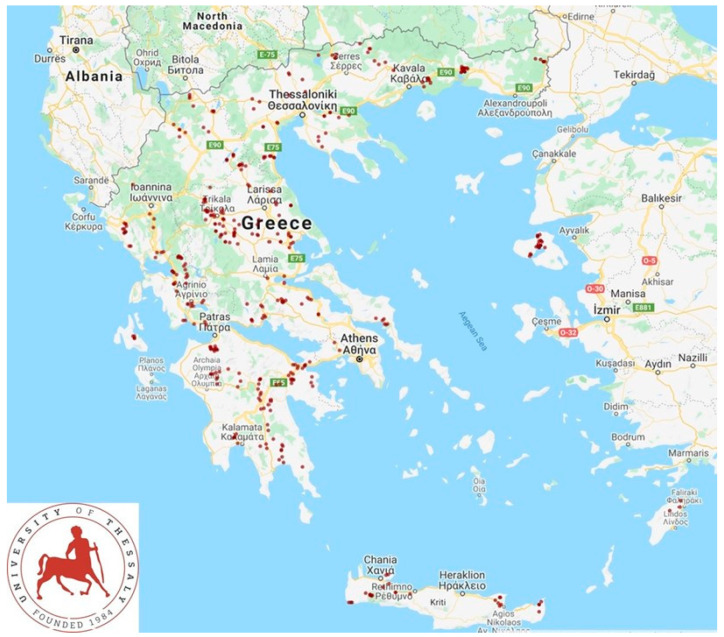
Location of 444 small-ruminant farms around Greece, which were visited for sampling of the bulk-tank raw milk for potential isolation of *Listeria* spp. (drawn with the use of GPS Visualizer (Adam Schneider; Portland, OR, USA)).

A cross-sectional study was performed from April 2019 to July 2020. In total, 325 sheep flocks and 119 goat herds in the 13 administrative regions of Greece (Figure 1) were included in the study and visited to collect samples and information. Veterinarians active in small ruminant health management around Greece were contacted by telephone and asked if they wished to collaborate in the investigation; in total, 48 veterinarians were contacted and of these, 47 (97.9%) agreed to collaborate. Farms were selected by the collaborating veterinarians on a convenience basis (the willingness of farmers to accept a visit by university personnel for an interview and sample collection) [21,22]. Visits were scheduled to 327 flocks, but on two occasions (0.6%), although the investigators had already arrived at these farms, the respective farmers refused to collaborate [21,22]. Three investigators (authors D.T.L., C.K.M. and G.C.F.) visited all the farms for sample collection. During the visit to each farm, data on the farm’s location were collected using hand-held Garmin global positioning system units. The geo-references were resolved to the specific farm level.

At the start of each visit, an interview of the farmer was performed by using a detailed questionnaire [23] to record management practices used for the flock. Next, samples were collected from the bulk-tank raw milk of the farms. The samples were collected directly from the milk cooling tank on each farm; all tanks had one or more agitators which stirred the milk continuously until the cover of the tank was raised for sample collection. Milk samples were collected in sterile plastic single-use pipettes which were immersed into the tank to withdraw the samples. In total, four 20 mL samples were collected from the milk tank of each farm, and a new pipette was used for each sample. Immediately after collection, samples were transferred into sterile plastic Universal-type vials.

Samples were stored at 0.0 to 4.0 °C using ice packs in portable refrigerators. Somatic cell counting and milk composition measurements were performed on each of the samples within 4 h after sample collection. Transportation of samples to the laboratory was undertaken by the investigators.

### 2.2. Overview of Laboratory Examinations

Of the four milk samples collected from the milk tank of each flock, two samples were used for somatic cell counting and milk composition measurements; the other two were used for the bacteriological examinations. With regard to the samples for bacteriological examination, two equal subsamples (i.e., 10 mL each) were created from each sample, i.e., in total, 4 subsamples of 10 mL each were available for all culturing experiments.

Somatic cell counts and milk composition measurements were taken within 4 h of collection as described before [21,22]. Each measurement was performed four times (i.e., once in each subsample derived from each original milk sample).

Bacteriological examinations started within 24 h of sample collection and included total bacterial counts, performed by following the standardized procedures described by Laird et al. [24], and culturing for the detection of *Listeria* spp. After completion of sample aliquot withdrawal for the microbiological examination, the temperature of the respective samples was measured and was found to never exceed 3.8 °C.

### 2.3. Procedures for Listeria *spp*. Isolation and Identification

The currently valid standardized protocol for *Listeria* spp. isolation ISO 11290-1:2017 [25] (title: Microbiology of the food chain—Horizontal method for the detection and enumeration of *Listeria monocytogenes* and of *Listeria* spp.) was applied for culturing samples for the isolation of *Listeria* spp. from the samples. For this work, each of the four subsamples from the bulk-tank raw milk was cultured independently. In brief, the procedure followed was as follows.

A total volume of 25 mL from each milk subsample (in equal proportions from each) was inoculated into 225 mL of half-Fraser broth, as appropriate, for incubation at 30 °C for 24 h (primary enrichment stage; half-Fraser broth is a modification of Fraser broth containing half of the concentration of nalidixic acid and acriflavine hydrochloride). Next, a volume (0.1 mL) of the cultured half-Fraser broth was inoculated, as appropriate, into Fraser broth (10 mL) for incubation at 37 °C for 48 h (secondary enrichment stage). The contents of the half-Fraser broth and the Fraser broth were separately plated onto (a) plates with *Listeria* agar acc. Ottaviani and Agosti and (b) plates with PALCAM *Listeria* selective agar for incubation at 37 °C for 48 h (culturing stage). The colonies on each plate were counted; if confluent growth was evident, this was marked as ‘+’ or ‘++’. The presence of at least one colony in any of the plates inoculated with material from each bulk-tank sample sufficed to indicate a suspicion of the presence of *Listeria* spp. All the colonies were subcultured onto blood agar for incubation at 37 °C for 48 h (refining stage) and were submitted for final identification.

The identification of all the colonies isolated on solid media was first performed by using conventional techniques. The identification was verified by means of matrix-assisted laser desorption/ionization time-of-flight mass spectrometry (MALDI TOF MS) (VITEK MS; BioMerieux, Marcy-l’-Étoile, France). In brief, each isolate was smeared from Petri dishes onto target slides, and then 1 μL of the VITEK MS matrix was applied over the sample, air-dried and allowed to co-crystallize with the sample; target slides with all isolates thus prepared were loaded into the VITEK MS system. Next, the mass spectra of whole bacterial cell proteins were acquired and compared with the known mass spectra included in the database for each species [26,27].

When a colony was not identified as *Listeria* sp., then another four colonies from the same agar plate were tested for confirmation of the negative result (unless, of course, fewer colonies were isolated, in which case all the colonies obtained were tested). All isolates confirmed as *Listeria* spp. were stored in brain–heart infusion broth with 15% glycerol at −80 °C.

### 2.4. Testing for Susceptibility to Antibiotics

All *Listeria* spp. isolates were tested for their susceptibility to antibiotics by means of the disk diffusion method, according to EUCAST recommendations (www.eucast.org; accessed on 15 February 2022). Briefly, for each isolate, a bacterial suspension with 1.5 × 10^8^ colony-forming units mL^−1^, which corresponded to 0.5 McFarland turbidity, was inoculated on Mueller–Hinton agar with 5% defibrinated horse blood and 20 mg L^−1^ *β*-nicotinamide adenine dinucleotide. Disks with benzylpenicillin (1 unit), ampicillin (2 μg), meropenem (10 μg), erythromycin (15 μg) and trimethoprim/sulfamethoxazole (1.25 μg/23.75 µg) were used for testing. The strain *Streptococcus pneumoniae* ATCC 49619 was used for quality control. The media were incubated for 24 h at 35 °C with 5% CO_2_. The zones of inhibition were measured (mm) and the isolates were categorized as susceptible or resistant according to the criteria of EUCAST for *Listeria monocytogenes*.

### 2.5. Data Management and Analysis

Data were entered into Microsoft Excel (Microsoft Corporation, Redmond, WA, USA) and analysed using SPSS v. 21 (IBM Analytics, Armonk, NY, USA). A basic descriptive analysis was performed. Exact binomial confidence intervals (CI) were obtained.

Initially, the potential association of isolation of *L. monocytogenes* or *L. ivanovii* from bulk-tank raw milk with somatic cell counts, total bacterial counts, the composition of bulk-tank raw milk and milk production on the farm was assessed by using one-way analysis of variance.

Next, 36 management-related and six human resource-related variables were evaluated for potential association with the isolation of *L. monocytogenes* or *L. ivanovii* from the bulk-tank raw milk of the farms (Table S2); these were either taken directly from the answers to the interview performed at the start of the visit or calculated from these answers. For each of these variables, categories were created according to the answers of the farmers.

Subsequently, climatic variables were derived from ‘The POWER (Prediction of Worldwide Energy Resources) Project’ (NASA Langley Research Center (LaRC), Hampton, VA, USA), which provides meteorological datasets from NASA research for supporting agricultural needs. The following settings were used for obtaining the data: user community, agroclimatology; temporal average, daily; latitude/longitude, geo-references of each farm; time extent, start date D16 to end date D1 (D0: day of the visit to each farm); output file format, ASCII. Data for nine climatic parameters were extracted (Table S2). For the evaluations, the daily results provided by this platform for the 15 days prior to the visit were averaged.

The outcomes of the isolation of *L. monocytogenes* from bulk-tank raw milk or the isolation of *L. ivanovii* from bulk-tank raw milk were considered. Exact binomial CIs were obtained. In the case of management-related and human resource-related variables, the importance of predictors was assessed by using cross-tabulation with Pearson’s chi-squared test and with simple logistic regression. In the case of climatic variables, the importance of the predictors was assessed by using analysis of variance. Separate univariate analyses were performed for each of the two outcomes. Subsequently, a multivariate model was created, initially including in the model all variables which achieved a statistical significance with a *p*-value of <0.20 in either of the two univariate analyses, i.e., for either of the two outcomes (*n* = 13). Variables were removed from the initial model by backward elimination. The *p*-value of the removal of a variable was assessed by the likelihood ratio test, and for those with a statistical significance with a *p*-value of >0.20, the variable with the largest probability was removed. This process was repeated until no variable could be removed with a value of >0.20. The variables required for the final multivariable model (*n* = 6) were: (a) number of female animals on the farm, (b) the presence of pigs on the farm, (c) the temperature of the cleaning water in the milking parlour, (d) the season of the start of the lambing period on the farm, (e) quarantining of new animals entering the farm and (f) average relative humidity during the 15 days prior to sampling. Finally, among farms with pigs, the numbers of these animals on the farms where *Listeria* spp. had been isolated were compared by using the Mann–Whitney test.

In all analyses, statistical significance was defined at *p* < 0.05.

### 2.6. Whole Genome Analysis of L. ivanovii Isolates

Whole genome analysis was performed for all *L. ivanovii* isolates obtained in the study. Initially, libraries were prepared using Ion Torrent technology and Ion Chef workflows (Thermo Fisher Scientific, Waltham, MA, USA). Sequencing was performed in the S5XLS system, and analysis of the primary data was conducted with Ion Torrent Suite v.5.10.0 (Thermo Scientific). The quality of the reads was checked using FastQC software [28]. The reads for each sample were assembled using the SPAdes genome assembler v3.15. 4 [29] with the default parameters. The quality of the assembled genomes was assessed with the tool Quast version 5.0.2 [30]. Each genome was annotated using Prokka version 1.14 [31] with the default parameters. The average coverage for each genome was computed using the tool bbmap (https://sourceforge.net/projects/bbmap/; accessed on 26 March 2022).

Evaluation for the presence of antibiotic resistance genes in the assembled genomes was performed by using the online tool ResFinder-4.1 [32,33,34], with the ID threshold set to 90% and the minimum length set to 60%. Evaluation for the presence of virulence factor genes was performed by using the online tool VirulenceFinder 2.0 [32,35,36], with the ID threshold set to 90% and the minimum length set to 60%. Subsequently, proteins encoded by the detected virulence factor genes were retrieved from the database of the National Center for Biotechnology Information. The function of each of these proteins was recorded in the UniProt Knowledge Base database (UniProtKB/Swiss-Prot (release 2022_01)).

The presence of mobile elements was assessed by using the tool Mobile Element Finder v1.0.3 [37]. The presence of prophages was assessed by using the tool PHASTER [38,39]. The presence of plasmids in the assemblies was assessed by means of Plasmid Finder v2.1 [32,40], using the Gram-positive bacteria database, with the minimum identity threshold set to 95% and the minimum coverage set to 60%. Lastly, the online tool CRISPRCasFinder was used, with the default parameters, to evaluate presence of CRISPR/CAS loci [41,42,43,44,45]. In order to determine the origin of the sequence of the spacers from the isolates obtained, a BLAST analysis was performed. Only results with a high identity score (100% coverage and ≥90% identity) were considered.

### 2.7. Phylogenetic Relationships of L. ivanovii Isolates

The phylogenetic relationships of the genomes of all *L. ivanovii* isolates obtained in the study were compared with genomes of *L. ivanovii* publicly available up to 15 March 2022 in GenBank [46]. In order to obtain the best possible phylogenetic resolution, the phylogenetic tree was constructed using the core genome of *L. ivanovii* [47]. For finding the core genome, the assembled genomes of *L. ivanovii* stored in the GenBank database were downloaded. Among these, two genomes with a very low N50 (GCA_003261115.1 with N50: 9946 and GCA_000183925.1 with N50: 3514) were excluded from this analysis. Next, by using the tool Prokka, the assemblies were annotated and, by using the CD-HIT tool [48,49], their proteins were grouped into groups of orthologous proteins (clusters) based on the similarity and coverage rates.

Two proteins were grouped in the same orthologous cluster if the threshold for similarity was over 75% for at least 70% of their length. Clusters in which all the genomes used in the analysis were found were considered to be the clusters of the core genome. In each cluster of the core genome, the different genes found in this cluster were aligned using the tool Clustal Omega [50,51,52]. Using the aligned data, the pseudogenome of every assembly was created; the pseudogenome of an assembly consisted of the aligned sequences of all the genes of this assembly contained in the clusters of the core genome, joined in series. Through a comparison of the different pseudogenomes, the phylogenetic tree of all *L. ivanovii* isolates in GenBank and of the *L. ivanovii* isolates obtained in the present study was created using the partitioned analysis of RAxML version 8.2.11 [53] with the GTRGAMMA model. As bootstrapping is computationally intensive, the RAxML’s rapid bootstrapping algorithm was used. Hence, 100 rapid bootstrap searches and 20 ML searches were conducted in order to find the best ML tree with its support values.

The best ML tree was edited in iTol software v 6.5.6 [54,55,56,57,58]. For each biosample, relevant information about the geographic location was extracted from GenBank information or the relevant literature and incorporated inτο the phylogenetic tree. From the best ML tree, using the online platform T-REX, we created the distance matrix of the *L. ivanovii* genomes [59]. Lastly, the BLAST Ring Image Generator (BRIG) was used to generate a circular map to compare the genomes of the strains sequenced in the present study with the strain PAM 55, an outbreak-related strain [60].

## 3. Results

### 3.1. Isolation of Listeria monocytogenes and Listeria ivanovii from Bulk-Tank Raw Milk

*L. monocytogenes* was isolated from the bulk-tank raw milk of one sheep farm; the isolation rate was 0.3% (1/325) (95% CI: 0.1–1.7%). *L. ivanovii* was isolated from the bulk-tank raw milk of three sheep farms; the isolation rate was 0.9% (3/325) (95% CI: 0.3–2.7%) (Table S3). The organisms were not isolated from the milk of goat farms; the isolation rate was 0.0% (0/119) (95% CI: 0.0–3.1%). There were no significant differences in the isolation rate between sheep and goat farms (*p* > 0.54 for all comparisons). *Listeria innocua*, *Listeria seeligeri* and *Listeria welshimeri* were not isolated from any sample.

There were no spatial associations among the four farms from which *L. monocytogenes* or *L. ivanovii* were isolated. Specifically, two of these farms were located in North Greece (in different administrative regions), one in Central Greece and one in the islands. The average distance between the three farms on the mainland was 191 km.

During susceptibility testing, the *L. monocytogenes* and all three *L. ivanovii* isolates were found to be phenotypically susceptible to all the antibiotics tested. The full results of susceptibility testing are in Table S4.

### 3.2. Lack of an Association of Isolation of L. monocytogenes or L. ivanovii with Milk Quality

There was no difference in somatic cell counts (*p* = 0.39), total bacterial counts (*p* = 0.66), fat content (*p* = 0.26) and protein content (*p* = 0.53) in the bulk-tank raw milk of farms from which *L. monocytogenes* or *L. ivanovii* was or was not isolated (Table S5). Moreover, there was no difference in the average milk quantity produced annually from ewes in flocks from which *L. monocytogenes* or *L. ivanovii* was or was not isolated (120 L, 233.9 ± 20.6 L and 207.5 ± 5.0 L, respectively; *p* = 0.61).

### 3.3. Predictors of the Isolation of L. monocytogenes or L. ivanovii from Bulk-Tank Raw Milk

**Figure 2:**
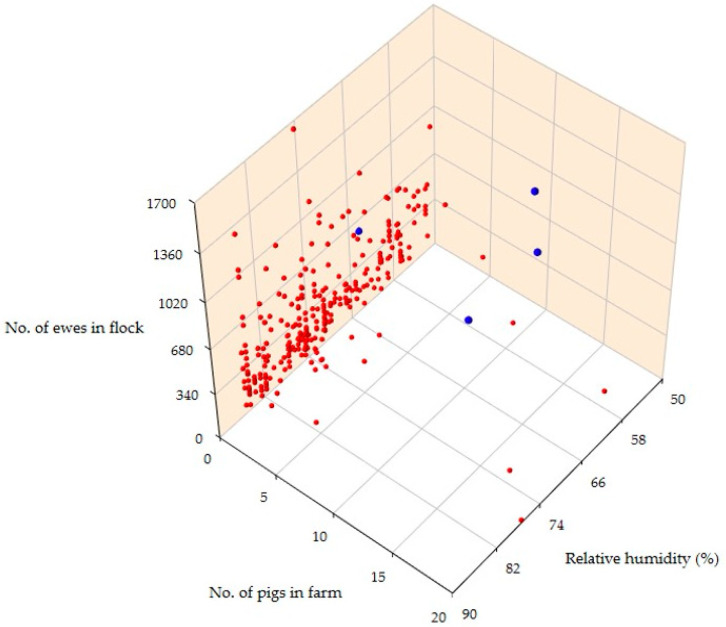
Scatterplot of predictors for the isolation of *L. monocytogenes* or *L. ivanovii* from the bulk-tank raw milk of sheep in Greece (red dots: farms from which no *L. monocytogenes* or *L. ivanovii* was isolated; blue dots: farms from which *L. monocytogenes* or *L. ivanovii* was isolated).

The details of the univariate analyses are in Tables S6–S8. In the multivariate analysis, three variables emerged as significant predictors for the isolation of *L. monocytogenes* or *L. ivanovii* from bulk-tank raw milk samples from sheep farms. These were: (a) the presence of pigs on the same farm (*p* < 0.0001), (b) low average relative humidity at 2 m (*p* = 0.006) and (c) a high number of female animals on the farm (*p* = 0.049) (Table 1, Figure 2).

Moreover, among farms with pigs, the farms where *L. monocytogenes* or *L. ivanovii* was isolated had a higher number of pigs (median: 10 (minimum, 10; maximum, 12)) than the farms where the bacteria were not isolated (two (1–20)) (*p* = 0.04) (Figure 1). An ad hoc inquiry with the veterinarians responsible for the three farms in which *L. monocytogenes* or *L. ivanovii* was isolated and where pigs were also present revealed that the pigs in these farms were fattening pigs (i.e., in anticipation for slaughter).

### 3.4. Whole Genome Analysis of L. ivanovii Isolates

The results of the whole genome analysis of the three *L. ivanovii* isolates are given in Table S9. No antibiotic resistance gene was found in any of the three genomes. Moreover, mobile elements, intact prophages and plasmids were also not found in any of the three assemblies (Table S10).

The following 15 virulence factor genes were detected in all three genomes: *flaA*, *lap*, oppA, *rli55*, *rli60*, *rsbv*, *clpp*, *degU*, *fri*, *hfq*, *inlC*, *lhrC*, *lhrC*, *lsp*, *tcsA* and *tig* (Tables S11 and S12). Finally, with regard to the CRISPR/CAS loci, four possible CRIPSRs and one CAS cluster were found in two assemblies (NGS01 and NGS03), and five CRISPRs and one CAS cluster in the third (NGS04) (Table S13). All three strains carried three identical CRISPR arrays (the same repeat and spacer sequences) and the same CAS cluster (CAS Type IB_1). In addition, the strains NGS03 and NGS04 shared a fourth common CRISPR array. BLASTn analysis showed that none of the spacers matched the phage genome.

### 3.5. Phylogenetic relationships of L. ivanovii Isolates

**Figure 3:**
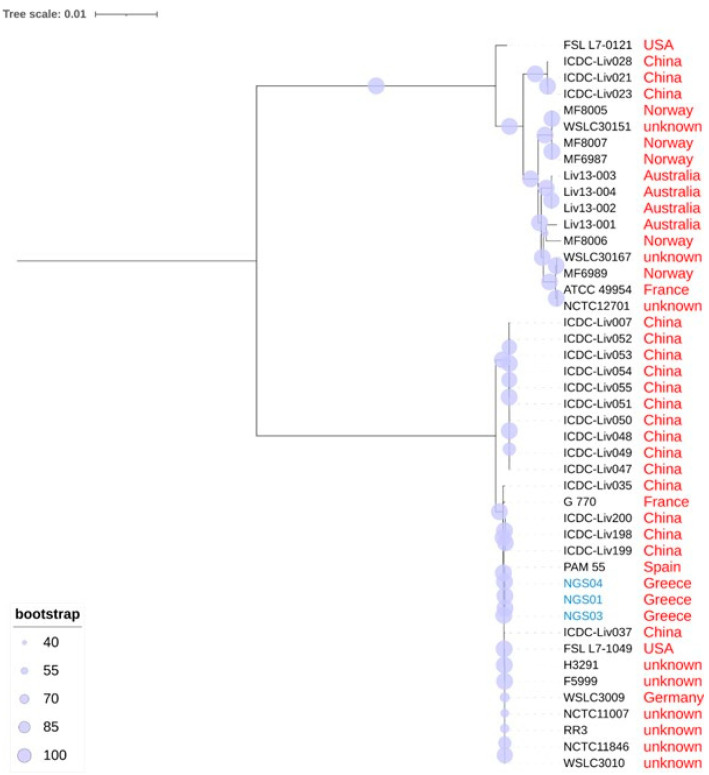
The maximum likelihood tree (GTRGAMMA model) of 45 *L. ivanovii* strains with bootstrapping support values (image created by Itol). Strains NGS01, NGS03 and NGS04, shown in blue typescript, were isolated from the bulk-tank raw milk of sheep during the present study.

The maximum likelihood tree (GTRGAMMA model) of 45 *L. ivanovii* strains with bootstrapping support values is shown in Figure 3. The tree was constructed by comparing 2042 genes, which form the core genome of *L. ivanovii.* The top branch of the tree includes 17 strains of *L. ivanovii* subsp. *londoniensis*; the lower branch includes 28 strains of *L. ivanovii* subsp. *ivanovii*. All three strains isolated in the present study were assigned to the *L. ivanovii* subsp. *ivanovii* branch (Figure 3).

The 28 *L. ivanovii* subsp. *ivanovii* strains form two clusters, one which includes only strains isolated in the People’s Republic of China and a second with strains from various other countries (including the three strains isolated in the present study) (Figure 3). The strains of that second cluster appear to be very similar to each other; the distance between them is small, despite the geographical diversity of the locations where these strains were isolated, which are from across various parts of the world (Table S14).

**Figure 4:**
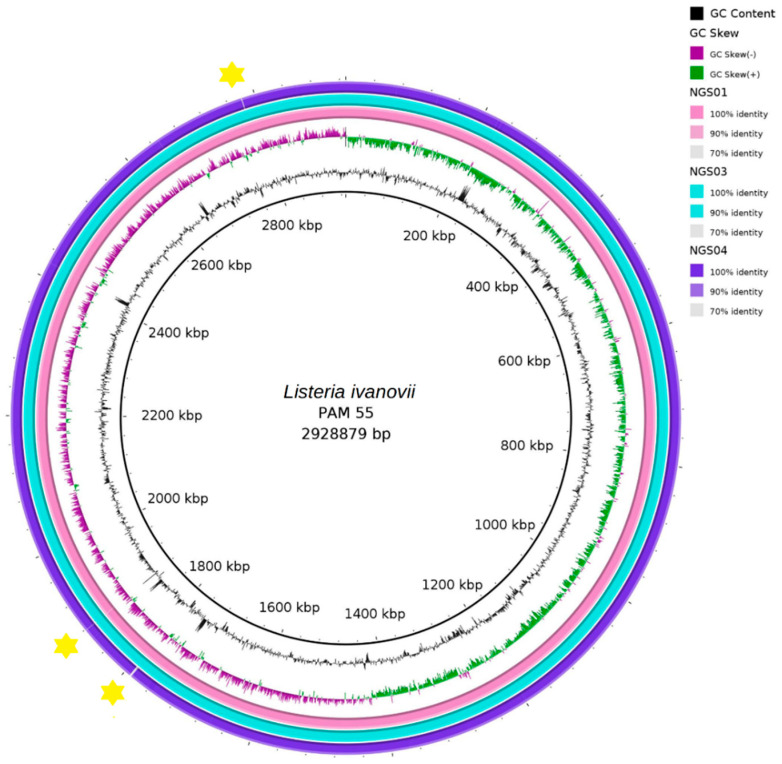
Comparison of the whole genome of three *L. ivanovii* strains (NGS01, NGS03, NGS04) isolated from the bulk-tank raw milk of sheep in Greece during the present study with the strain PAM 55, isolated in 1997 in Spain from an outbreak of abortion in sheep, using the tool BRIG; the yellow asterisks indicate the regions where the similarities of the NGS01, NGS03, NGS04 strains to PAM 55 were below 70%.

The three strains isolated in the present study were found to be phylogenetically close to the PAM 55 strain, which was isolated in 1997 in Spain from an outbreak of abortion in sheep (Figure 3, Table S14). Indeed, the three genomes were found to have many similarities to that of the PAM 55 strain, with only minor differences identified in a few regions, e.g., in the region near 1800 kbp (Figure 4).

## 4. Discussion

### 4.1. Presence and Identification of L. monocytogenes or L. ivanovii in Bulk-Tank Milk

The results provide evidence regarding the low prevalence of *Listeria* spp. in milk samples from dairy sheep and goat farms in Greece. In the present study, sheep and goat farms from all regions of Greece were included in the study; that way, conditions prevailing throughout the country were taken into account and factors of regional importance weighed less. In order to minimize possible bias, the study also used consistent methodologies and ensured that specific tasks were always performed by the same investigators.

We note that *L. ivanovii* was detected for the first time in Greece in a large collection of the milk of sheep and goats. During the whole genome sequencing, which was followed by a phylogenetic analysis, the strains were identified as *L. ivanovii* subspecies *ivanovii*. This could not be determined by the use of MALDI TOF MS alone, despite its high discriminatory capacity.

These findings are, in general, similar to those found in other countries of Europe [61]. For example, Amagliani et al. [62] and Condoleo et al. [63] did not isolate the pathogens from samples of raw sheep milk in Italy, and Bogdanovicova et al. [64] isolated only *L. monocytogenes* (<4% of samples) in a similar investigation in the Czech Republic; however, in sharp contrast, Artursson et al. [65] reported an isolation rate of 29% from raw sheep and goat milk samples in Sweden. Notably, in all previous relevant studies, the isolation rate of *L. monocytogenes* was generally higher than that of *L. ivanovii* [66,67].

Despite the low rate of isolation of the pathogens in the raw milk, there was a large variation in the isolation rates of *Listeria* spp. pathogens in samples from cheese of milk of sheep or goat origin in Greece. In recent studies, these were found to vary from 0% [68,69] to 39% [70] of samples tested. Potential sources of *Listeria* spp. in cheese, apart from the raw material (i.e., the milk), also include the environment of the processing plants [71,72]. Given the low rate of isolation of the pathogens in raw milk, close monitoring of cheese production plants is necessary to prevent contamination by the bacteria.

In any case, the mean annual notification rate of human listeriosis in Greece was 1.03 cases per 1,000,000 population for the period 2004–2017. Apparently, this is lower than the respective rate in EU countries (for 2016: 1.85 and 4.7 cases per 1,000,000 population) [73]. To our knowledge, only *L. monocytogenes* isolates have been identified from clinical samples from people in Greece.

### 4.2. Predictors of the Ιsolation of L. monocytogenes or L. ivanovii in Βulk-Τank Μilk

Reports of the isolation of *Listeria* spp. in clinical samples from pigs are infrequent for both *L. monocytogenes* [74,75] and *L. ivanovii* [76,77]. Hence, the identification of the presence of pigs in a farm as a predictor of the isolation of *L. monocytogenes* or *L. ivanovii* in bulk-tank milk was surprising.

Although clinical listeriosis is rare in pigs [78,79,80], domestic pigs could be a possible reservoir of *Listeria* spp. [81,82]. *Listeria* spp. has been occasionally isolated from faecal and skin samples of clinically healthy pigs [83]. The organism is considered to be harboured in the intestinal tract of these animals, and the prevalence of the isolation of *L. monocytogenes* from faecal samples ranges from 0% to 47% [84]. We hypothesise that, possibly, pigs might have been infected subclinically, excreting the pathogens in their faeces, thus contaminating the environment and contributing to an increased risk of infection of the grazing ewes, which subsequently excreted the organism in their faeces or the milk. However, the possibility that the teats of ewes might have been in touch with the contaminated ground of the farm and thus become contaminated with *L. monocytogenes* or *L. ivanovii* cannot be excluded. In the affected sheep farms, the pigs were maintained mainly to provide food for the family rather than for commercial purposes; in these circumstances, pigs are often allowed increased access to outdoor areas, which can contribute to contamination of the farm environment. In fact, in previous studies, the isolation of *Listeria* spp. from paddocks where pigs and cattle were co-grazing has been reported [76]. The association of the number of pigs on the farm with the isolation of the bacteria lends further support to this hypothesis, as a larger number of infected pigs would have contributed to higher levels of contamination of the grazing areas.

Moreover, the identification of the number of ewes in the flock as another predictor of the isolation of *L. monocytogenes* or *L. ivanovii* from milk may also be linked to the increased burden of the bacteria in the flock. Consequently, larger amounts of the bacteria are excreted in the milk, making the isolation easier.

The emergence of relative humidity as a predictor of the isolation of the organism can be possibly related to the growth characteristics of the organism. The results of previous studies regarding the potential effects of relative humidity on *L. monocytogenes* are conflicting, whilst no relevant studies for *L. ivanovii* are available. In food-related matrices, relative humidity in the range from 43% to 68% was found to adversely affect the viability of the bacteria [85,86] as the result of changes in the permeability of the bacterial membrane [86]. In contrast, in an experimental study that assessed viability of *L. monocytogenes* in the environment, this was found to be best at a relative humidity of around 50% (i.e., close to the mean relative humidity of the four farms where *L. monocytogenes* or *L. ivanovii* was isolated, which was 55.5%), irrespective of the temperature [87]. One should also consider that perhaps reasons unrelated to the bacteria themselves might have also played a role; for example, under conditions of lower relative humidity, sheep feel more comfortable and are more likely to be outdoors for grazing, which can contribute to spread of the bacteria within the flock.

### 4.3. Bioinformatics Assessment

The core genome of a bacterial species consists of the genes that are reserved in all the strains of the species, suggesting that these are probably essential for the survival and growth of the species [88]. There is no consensus on the procedure used to find the core genome’s genes, and different approaches exist in the literature. In the present analysis, we considered the core genome genes to be the protein coding genes, which were found to be present in all the strains studied. Phylogenetic analysis using the core genome genes can provide a deep and detailed phylogeny of the analysed bacteria. The core genome’s phylogenetic analysis outperformed phylogeny using 16S rRNA gene, as the core genome’s phylogeny acknowledges multiple conserved genes in the analysed species.

In the present analysis, the core genome phylogenetic tree correctly divided *L. ivanovii* into the two known subspecies, *L. ivanovii* subsp. *ivanovii* and *L. ivanovii londoniensis*, and also indicated the two distinct branches of *L. ivanovii* subsp. *ivanovii*. Gan et al. [89] indicated that strains from these two branches differed in motility, metabolic activity and virulence. The strains within the second branch, where the three strains of *L. ivanovii* isolated in the present study (NGS01, NGS03 and NGS04) have been assigned, exhibit a very small evolutionary distance, even though they are globally dispersed. This suggests either that the global spread of this specific clonal branch is a recent evolutionary event or that this branch is characterized by a relatively slow evolutionary rate.

Moreover, the three strains of *L. ivanovii* isolated in the present study have been analysed in silico for various genetic and phenotypic traits in order to fully assess them. Antibiotic resistance genes have been detected in none of these strains, which was consistent with the results of susceptibility testing. The strains also have not been found to have any plasmid or intact prophages. Incomplete prophages probably cannot perform the essential phage functions [90], which might have contributed to the lack of antibiotic resistance.

The results of the BLASTn analysis of the spacers in the CRISPR array may possibly explain the fact that *L. ivanovii* subsp. *ivanovii* strains are sensitive to bacteriophages, whereas the other subspecies seem to be resistant to infection by phages [91].

During the analysis, 15 virulence factor genes were also detected, and the three strains all contained the exact same genes. Among these, the presence of *flaA* and *degU* suggest the motility of these strains [89,92], whilst *inlC* and *lap* encode for factors that participate in the pathogenicity of *L. monocytogenes* or *L. ivanovii* strains [93,94]. Flagellin A, encoded by *flaA*, is considered to play a role in actin polymerization, which occurs at nucleation sites on the surface of the pathogen, to form the filaments of the flagella of *Listeria* spp. These are nucleated on the surface of the organism, possibly as branches from the surrounding cytoskeletal filaments in a way that, finally, the comet tail of the pathogen consists of a stiff scaffold of interconnected bundles [95]. Once the comet tail is longer than the organism itself, the pathogen can move while the comet tail remains stationary [96]. Nevertheless, none of the three strains harboured genes for *Listeria* pathogenicity island 1 and *Listeria* pathogenicity island 2, which, according to Gan et al. [89], are the predominant factors in the pathogenicity of *L. ivanovii*. This may possibly explain the lack of differences in the quality of milk produced from the farms from which the organism was isolated, as these strains lacked the increased pathogenicity causing mammary lesions in the affected ewes.

Despite the above, these three strains are evolutionarily close to the strains PAM 55 and G770, which were isolated from an outbreak of abortion in sheep in Spain [4] and a fatal case of aortic prosthesis infection in France [97], respectively, and thus should be monitored, given the importance of the small ruminant industry in the country and the increased production of cheese from the milk of these animals. The epidemiological surveillance of bacteria isolated from diverse environmental sources can help in preventing the emergence of hypervirulent and super-resistant isolates.

## 5. Conclusions

This work describes a comprehensive assessment of a little-studied zoonotic pathogen. The infrequent isolation of *Listeria ivanovii* from samples from small-ruminant farms, linked with the lack of isolation from dairy products, indicates a small risk for people consuming such products. The identification of predictors of the isolation of the organism should be taken into account in the health management of farms. The findings of the molecular studies, taken together, suggest that possibly, the reduced evolutionary diversity of the three strains is reflected in the reduced pathogenicity of the organism.

## Figures and Tables

**Table 1:** Results of the multivariate analysis for the isolation of *L. monocytogenes* or *L. ivanovii* from the bulk-tank raw milk of sheep farms during a countrywide investigation in Greece.

Variables (*n* = 3)	Odds Ratios ^1^ (95% CI)	*p*
Isolation of *L. monocytogenes*		
Presence of pigs on the farm		<0.0001
Yes (1/36 = 2.8%)	24.46 (0.98–612.06)	0.05
No (0/289 = 0.0%)	reference	-
Average relative humidity at 2 m		0.006
Per unit decrease	6.09 (0.00–13.19)	0.006
Number of female animals on the farm		
≤165 (0/88 = 0.0%)	1.36 (0.03–69.29)	0.88
166–330 (0/120 = 0.0%)	reference	-
331–500 (0/66 = 0.0%)	1.81 (0.04–92.38)	0.77
>500 (1/51 = 2.0%)	7.16 (0.2 9–178.71)	0.23
Isolation of *L. ivanovii*		
Presence of pigs on the farm		<0.0001
Yes (2/36 = 5.6%)	17.00 (1.50–192.46)	0.022
No (1/289 = 0.3%)	reference	-
Average relative humidity at 2 m		0.006
Per unit decrease	6.09 (0.00–13.19)	0.006
Number of female animals on the farm		0.049
≤165 (0/88 = 0.0%)	1.36 (0.03–69.29)	0.88
166–330 (0/120 = 0.0%)	reference	-
331– 500 (1/66 = 1.5%)	5.52 (0.22–137.41)	0.30
>500 (2/51 = 3.9%)	12.17 (0.57–258.14)	0.11

^1^ Odds ratio calculated against the associations with the lowest prevalence of the variable.

## Data Availability

Most data presented in this study are in the Supplementary Materials. The remaining data are available on request from the corresponding author. Some of the data are not publicly available as they form part of the PhD thesis of the first author, which has not yet been examined, approved and uploaded in the official depository of PhD theses from Greek universities. The accession number of the Bioproject deposited in the NCBI database is PRJNA812022. In the Bioproject record, there are links to the biosamples (SAMN26366399, SAMN26366413, SAMN26366412) and to the Sequence Read Archive (SRA) data (SRS12212767, SRS12212768, SRS12212769). The genomes of the *L. ivanovii* strains NGS01, NGS03 and NGS04 isolated in the present study were deposited in GenBank under accession numbers GCA_022815685.1, GCA_022815695.1 and GCA_022815665.1, respectively.

## Supplementary Materials

The following supporting information can be downloaded at: https://www.mdpi.com/article/10.3390/biology11060871/s1. Table S1: Classification of articles found in the Web of Science presenting work related to *Listeria ivanovii*, according to the topic presented therein and the origin of the study (by country). Table S2: Variables evaluated for potential association with the isolation of *Listeria monocytogenes* and *Listeria ivanovii* from the bulk-tank raw milk of 444 small-ruminant farms during a countrywide investigation in Greece. Table S3: Details of the isolation of *Listeria monocytogenes* and *Listeria ivanovii* from the bulk-tank milk raw milk of 325 sheep farms during a countrywide investigation in Greece. Table S4: Details of the results of testing (diameters of the zones of inhibition) for susceptibility to antibiotics in *Listeria monocytogenes* and *Listeria ivanovii* from the bulk-tank raw milk of 325 sheep farms during a countrywide investigation in Greece. Table S5: Quality parameters of bulk-tank raw milk of 325 sheep farms from which *Listeria monocytogenes* and *Listeria ivanovii* were or were not isolated during a countrywide investigation in Greece. Table S6: Results of the univariate analysis for the association of husbandry-related variables with the isolation of *Listeria monocytogenes* or *Listeria ivanovii* from the bulk-tank raw milk of 325 sheep farms during a countrywide investigation in Greece. Table S7: Results of the univariate analysis for the association of human resource-related variables with the isolation of *Listeria monocytogenes* or *Listeria ivanovii* from the bulk-tank raw milk of 325 sheep farms during a countrywide investigation in Greece. Table S8: Results of univariate analysis for the association of climate-related variables with the isolation of *Listeria monocytogenes* or *Listeria ivanovii* from the bulk-tank raw milk of 325 sheep farms during a countrywide investigation in Greece. Table S9: Metrics of the assembled genome of three *L. ivanovii* isolates from the bulk-tank raw milk of sheep in Greece. Table S10: Summary findings of the assessment of genomes, using the tool PHASTER, of three *Listeria ivanovii* isolates obtained from the bulk-tank raw milk of sheep in Greece for the presence of prophages. Table S11: Virulence factor genes detected by using the tool VirulenceFinder 2.0 in the genome of three *Listeria ivanovii* isolates obtained from the bulk-tank raw milk of sheep in Greece. Table S12: Proteins encoded by the virulence factor genes (in alphabetical order) detected in the genome of three *Listeria ivanovii* isolates obtained from the bulk-tank raw milk of sheep in Greece. Table S13: The output of CRISPRCasFinder for the genome of three *Listeria ivanovii* isolates obtained from the bulk-tank raw milk of sheep in Greece. Table S14: Distance matrix between the 45 *Listeria ivanovii* strains included in the maximum likelihood tree.
