# Supplementary material for: Isolation of Listeria ivanovii from Bulk-Tank Milk of Sheep and Goat Farms—From Clinical Work to Bioinformatics Studies: Prevalence, Association with Milk Quality, Antibiotic Susceptibility, Predictors, Whole Genome Sequence and Phylogenetic Relationships
Source: Biology (Basel). 2022 Jun 6;11(6):871. doi: 10.3390/biology11060871 (PMC9220212; doi:10.3390/biology11060871)
Supplement: Supplementary file 1 [file biology-11-00871-s001.zip › biology-1731840-Supplementary Tables S1-S13.pdf]

Isolation of *Listeria ivanovii* from bulk-tank milk of sheep and goats farms: prevalence, association with milk quality, antibiotic susceptibility, predictors, whole genome sequence and phylogenetic relationships

Daphne T. Lianou†, Anargyros Skoulakis†, Charalambia K. Michael, Eleni I. Katsarou, Dimitrios C. Chatzopoulos, Nikolaos Solomakos, Katerina Tsilipounidaki, Zoe Florou, Peter J. Cripps, Angeliki I. Katsafadou, Natalia G.C. Vasileiou, Konstantina S. Dimoveli, Maria V. Bourganou, Dimitra V. Liagka, Vasileios G. Papatsiros [1], Panagiota I. Kontou, Vasia S. Mavrogianni, Mariangela Caroprese, Efthymia Petinaki, George C. Fthenakis

† These authors have contributed equally and their names are listed alphabetically.

Table S1. Classification of articles found in Web of Science presenting work related to *Listeria ivanovii,* in accord with the topic presented therein and the origin of the study (by country).

				n	
**Total number of articles**				**355**	
1. Characteristics of the organism				103	
2. Isolation of the organism from samples from foods				92	
2.1. Isolation from meat and meat products				34	
2.2. Isolation from dairy products				23	
2.3. Isolation from fish and seafood				15	
2.4. Isolation from vegetables				11	
2.5. Isolation from ready-to-eat foods				9	
2.6. Isolation of tomatoes				2	
2.7. Isolation from fruits				1	
2.8. Isolation from infant food				1	
2.9. Isolation from pet snacks				1	
2.10.Isolation from other foods				2	
2.A. Origin (by country) of the 92 articles referring to isolation of the organism from samples from foods					
Algeria	1	Australia	1	Brazil	1
Bulgaria	2	Canada	2	PR China	2
Colombia	1	Costa Rica	1	Egypt	2
Ethiopia	1	Germany	3	Greece	1
India	4	Iran	7	Ireland	1
Italy	5	Jordan	5	Malaysia	3
Mexico	3	Nigeria	3	Portugal	1

Saudi Arabia	1	Rep. South Africa	2	South Korea	2
Spain	8	Sudan	1	Taiwan	1
Thailand	3	Trinidad & Tobago	1	Turkey	9
United Kingdom	1	United States of America	9	Venezuela	4
3. Diagnostics				72	
4. Isolation of the organism from clinical samples from animals				37	
4.1. Isolation from clinical samples from sheep				12	
4.2. Isolation from clinical samples from cattle				8	
4.3. Isolation from clinical samples from buffaloes				3	
4.4. Isolation from clinical samples from rodents				3	
4.5. Isolation from clinical samples from terrestrial wildlife animals				3	
4.6. Isolation from clinical samples from goats				2	
4.7. Isolation from clinical samples from pigs				2	
4.8. Isolation from clinical samples from poultry				2	
4.9. Isolation from clinical samples from chinchilla				1	
4.10. Isolation from clinical samples from guinea pigs				1	
4.11. Isolation from clinical samples from sea mammals				1	
4.12. Isolation from clinical samples from zoo animals				1	
4.A. Origin (by country) of articles referring to isolation of the organism from clinical samples from animals					
Australia	2	Belgium	2	Egypt	1
France	1	Germany	3	India	12
Iran	1	Israel	1	Kenya	1
Poland	1	Spain	4	Switzerland	1
Turkey	3	United Kingdom	3	United States of America	1
4.12. Isolation from clinical samples from humans				6	
4.B. Origin (by country) of articles referring to isolation of the organism from clinical samples from people					
France	2	Germany	1	Israel	1
Spain	1	United Kingdom	2		
5. Isolation of the organism from environmental samples				19	
5.1. Isolation from samples from food-related environments				6	
5.A. Origin (by country) of articles referring to isolation of the organism from samples from food-related environments					
Colombia	1	India	1	Ireland	1
Italy	1	Portugal	1	Slovakia	1
5.2. Isolation from samples from non- food-related environments				13	

5.B.	Origin (by country) of articles referring to isolation of the organism from samples from non-food-related environments				
Austria	1	France	1	Greece	1
India	1	Kenya	1	Mexico	1
Nigeria	1	Rep. South Africa	2	Spain	1
Switzerland	1	United Kingdom	1	United States of America	1

6.	Pathogenesis studies		17
	6.1.	Studies in mice	9
	5.2.	Studies in sheep	2
	5.3.	Studies in cattle	1
	5.4.	Studies in rabbit	1
	5.5.	Studies in zebrafish	1
	5.6.	Studies in material from humans	5
7.	Protection of foods		14
8.	Various topics		5

Table S2. Variables evaluated for potential association with isolation of *Listeria monocytogenes* and *Listeria ivanovii* from bulk-tank raw milk of 444 small ruminant farms during a countrywide investigation in Greece.

**Husbandry-related variables (*n* = 36)**
Management system applied in the farm (description according to EFSA classification [1])
Month into the lactation period at sampling (month)
No. of female animals in the farm (no.)
Presence of cattle or buffaloes in the farm (yes / no)
Presence of pigs in the farm (yes / no)
Presence of rabbits in the farm (yes / no)
Presence of poultry in the farm (yes / no)
Presence of dogs in the farm (yes / no)
Presence of cats in the farm (yes / no)
Presence of exotic animals in the farm (yes / no)
Presence of equids in the farm (yes / no)
Presence of rodents in the farm (yes / no)
Grazing of wildlife animals in same areas as animals of the farm (yes / no)
Machine- or hand-milking (yes / no)
Temperature of cleaning water in the milking parlour (^oC)
Use of detergent for parlour cleaning after the milking sessions (yes / no)
Temperature of milk storage tank (^oC)
Total milk quantity per ewe / doe obtained during the preceding milking period (litres)
Start of lambing / kidding period in the farm (all year / autumn / winter / spring or summer)
Preparation of dairy products for home consumption at the farm (yes / no)
Collaboration with a veterinarian (yes / no)
Total visits made annually by veterinarians to the farm during the preceding season (no. of visits)
Application of quarantine in new animals entering into the farm (yes / no)
Transhumance practiced in the farm (yes / no)
Disinfections performed in the farm (yes / no)
Annual frequency of systemic disinfections in the farm (no.)
Daily number of milking sessions (no.)
Use of teat disinfection after milking
Grazing practice (yes / no)
Grazing land available to animals (acres per animal)
Annual duration of grazing (months)
Availability of straw bedding (yes / no)
Provision of hay as fodder to animals (yes / no)
Average quantity of hay provided daily to animals during the preceding season (kg)
Provision of silage to adult animals (yes / no)

Provision of finished feed (concentrate) to adult animals (yes / no)
**Human resources-related variables (*n* = 6)**
Age of farmer (years)
Length of previous animal farming experience (years)
General education (description)
Farmer by profession (yes / no)
Family tradition in farming (yes / no)
Presence of working staff in the farm (yes/no)
**Climate-related variables (*n* = 9)**
Average precipitation during the 15 days prior to sampling (mm)
Average relative humidity during the 15 days prior to sampling (%)
Average minimum temperature at 2 m during the 15 days prior to sampling (^{o}C)
Average maximum temperature at 2 m during the 15 days prior to sampling (^{o}C)
Average temperature range at 2 m during the 15 days prior to sampling (^{o}C)
Average mean temperature at 2 m during the 15 days prior to sampling (^{o}C)
Average earth skin temperature at 2 m during the 15 days prior to sampling (^{o}C)
Average wind speed at 10 m during the 15 days prior to sampling (m s^{-1})
Average all sky insolation incident during the 15 days prior to sampling (Wh m-2)

[1] management system classified as intensive, semi-intensive, semi-extensive, extensive (European Food Safety Authority. Scientific opinion on the welfare risks related to the farming of sheep for wool, meat and milk production. *EFSA J.* 2014;12:3933–4060).

Table S3. Details of isolation of *Listeria monocytogenes* and *Listeria ivanovii* from bulk-tank milk raw milk of 325 sheep farms during a countrywide investigation in Greece.

	**Isolation from plates cultured from inoculated half-Fraser broth**		**Isolation from plates cultured from inoculated Fraser broth**	
Isolate reference	Plates with *Listeria* agar acc. Ottaviani and Agosti	Plates with PALCAM *Listeria* selective agar	Plates with *Listeria* agar acc. Ottaviani and Agosti	Plates with PALCAM *Listeria* selective agar
		L. monocytogenes		
NGS02 [1]	+ growth	++ growth	+ growth	+ growth
	+ growth	++ growth	+ growth	+ growth
	+ growth	++ growth	+ growth	++ growth
	+ growth	++ growth	+ growth	+ growth
		L. ivanovii		
NGS01	no growth	no growth	5 colonies	9 colonies
	no growth	no growth	4 colonies	10 colonies
	no growth	no growth	5 colonies	6 colonies
	no growth	no growth	7 colonies	8 colonies
NGS03	no growth	+ growth	+ growth	+ growth
	no growth	+ growth	+ growth	++ growth
	no growth	+ growth	+ growth	++ growth
	no growth	+ growth	+ growth	+ growth
NGS04	no growth	++ growth	50 colonies	++ growth
	no growth	++ growth	43 colonies	++ growth
	no growth	++ growth	62 colonies	++ growth
	no growth	++ growth	53 colonies	++ growth

[1] results of the four sub-samples from bulk-tank raw milk are presented for each isolate.

Table S4. Details of results of testing (diametres of zones of inhibition) for susceptibility to antibiotics of *Listeria monocytogenes* and *Listeria ivanovii* from bulk-tank milk raw milk of 325 sheep farms during a countrywide investigation in Greece.

	Antibiotics used for testing (diametre of inhibition zone for characterisation as sensitive (S) or resistant (R))				
Isolate reference	Ampicillin (S ≥ 16, R < 16 mm [1])	Benzylpenicillin (S ≥ 13, R < 13 mm)	Erythromycin (S ≥ 25, R < 25 mm)	Meropenem (S ≥ 26, R < 26 mm)	Trimethoprim – sulfamethoxazole (S ≥ 29, R < 29 mm)
L. monocytogenes					
NGS02 [1]	20 mm	18 mm	30 mm	29 mm	33 mm
L. ivanovii					
NGS01	21 mm	18 mm	30 mm	30 mm	34 mm
NGS03	21 mm	18 mm	30 mm	30 mm	34 mm
NGS04	21 mm	18 mm	30 mm	30 mm	34 mm

[1] S: isolate sensitive, R: isolate resistant.

Table S5. Quality parameters of bulk-tank raw milk of 325 sheep farms from which *Listeria monocytogenes* and *Listeria ivanovii* was or was not isolated during a countrywide investigation in Greece.

	**Somatic cell counts (cells mL^{-1})**	**Total bacterial counts (cfu mL^{-1})**	**Fat content (%)**	**Total protein content (%)**
Isolation of *L. monocytogens* from the bulk-tank raw milk (*n* = 1)	0.690×10^6	57×10^3	5.72	4.52
Isolation of *L. ivanovii* from the bulk-tank raw milk (*n* = 3)	0.339×10^6 (95% CI: 0.218×10^6 - 0.526×10^6)	264×10^3 (95% CI: 51×10^3 - 1349×10^3)	6.72 ± 0.07	4.33 ± 0.09
No isolation of *L. ivanovii* or *L. monocytogens* from the bulk-tank raw milk (*n* = 321)	0.486×10^6 (95% CI: 0.448×10^6 - 0.526×10^6)	402×10^3 (95% CI: 339×10^3 - 0.479×10^3)	6.16 ± 0.05	4.43 ± 0.01
p	0.39	0.66	0.26	0.53

Table S6. Results of univariable analysis for association of husbandry-related variables with isolation of *Listeria monocytogenes* or *Listeria ivanovii* from bulk-tank raw milk of 325 sheep farms during a countrywide investigation in Greece.

**Isolation of *L. monocytogens* from the bulk-tank raw milk (n = 1)**				**Isolation of *L. ivanovii* from the bulk-tank raw milk (n = 3)**				**No isolation of *L. ivanovii* or *L. monocytogens* from the bulk-tank raw milk (n = 321)**				p^{1}	p^{2}
**Management system applied in the flock**													
Intensive	Semi-intensive	Semi-extensive	Extensive	Intensive	Semi-intensive	Semi-extensive	Extensive	Intensive	Semi-intensive	Semi-extensive	Extensive		
0	1	0	0	0	1	2	0	43	149	105	24	0.76	0.62
**Month into the lactation period at sampling**													
0–1st	2nd–5th	6th–9th	After 9th	0–1st	2nd–5th	6th–9th	After 9th	0–1st	2nd–5th	6th–9th	After 9th		
0	0	1	0	0	0	3	0	23	138	143	17	0.75	0.30
**No. of female animals in the farm**													
≤ 165 ewes	166-330 ewes	331-500 ewes	> 500 ewes	≤ 165 ewes	166-330 ewes	331-500 ewes	> 500 ewes	≤ 165 ewes	166-330 ewes	331-500 ewes	> 500 ewes		
0	0	0	1	0	0	1	2	88	120	65	48	0.15	0.07
**Presence of cattle or buffaloes in the farm**													
Yes		No		Yes		No		Yes		No			
0		1		0		3		33		288		0.74	0.56
**Presence of pigs in the farm**													
Yes		No		Yes		No		Yes		No			
1		0		2		1		33		288		0.005	0.002
**Presence of rabbits in the farm**													
Yes		No		Yes		No		Yes		No			
0		1		0		3		28		293		0.76	0.59
**Presence of poultry in the farm**													
Yes		No		Yes		No		Yes		No			
0		1		1		2		180		141		0.26	0.43
**Presence of dogs in the farm**													
Yes		No		Yes		No		Yes		No			
1		0		3		0		293		28		0.76	0.59
**Presence of cats in the farm**													
Yes		No		Yes		No		Yes		No			
1		0		3		0		167		154		0.34	0.10

**Presence of exotic animals in the farm**													
Yes	No	Yes	No	Yes	No								
0	1	0	3	0	321							n/a	n/a
**Presence of equids in the farm**													
Yes	No	Yes	No	Yes	No								
0	1	0	3	57	264							0.64	0.42
**Presence of rodents in the farm**													
Yes	No	Yes	No	Yes	No								
1	0	3	0	321	0							n/a	n/a
**Grazing of wildlife animals in same areas as animals of the farm**													
Yes	No	Yes	No	Yes	No								
1	0	0	3	151	170							0.29	0.10
**Machine- or hand-milking**													
Machine-milking	Hand-milking	Machine-milking	Hand-milking	Machine-milking	Hand-milking								
1	0	3	0	251	70							0.60	0.36
**Temperature of cleaning water in the milking parlour**													
≤ 75 °C	> 75 °C	≤ 75 °C	> 75 °C	≤ 75 °C	> 75 °C								
1	0	3	0	152	99							0.42	0.17
**Use of detergent for parlour cleaning after the milking sessions**													
Yes	No	Yes	No	Yes	No								
1	0	3	0	248	3							0.91	0.85
**Temperature of milk storage tank**													
≤ 4 °C	> 4 °C	≤ 4 °C	> 4 °C	Yes	No								
1	0	3	0	231	71							0.58	0.34
**Total milk quantity per ewe obtained during the preceding milking period**													
≤ 200 L	201-400 L	> 400 L	≤ 200 L	201-400 L	> 400 L	≤ 200 L	201-400 L	> 400 L					
1	0	0	0	3	0	172	134	15				0.64	0.13
**Start of lambing period in the farm**													
All year	Autumn	Winter	Spring or summer	All year	Autumn	Winter	Spring or summer	All year	Autumn	Winter	Spring or summer		
0	1	0	0	1	2	0	0	17	242	55	7	0.21	0.18
**Preparation of dairy products for home consumption**													
Yes	No	Yes	No	Yes	No								
0	1	1	2	200	121							0.20	0.31

**Collaboration with a veterinarian**													
Yes	No	Yes	No	Yes	No								
1	0	3	0	273	48							0.68	0.47
**Total visits made annually by veterinarians to the farm during the preceding season**													
≤ 4	5 - 7	> 7	≤ 4	5 - 7	> 7	≤ 4	5 - 7	> 7					
1	0	0	1	1	1	137	85	99				0.51	0.94
**Application of quarantine in new animals entering into the farm**													
Yes	No	Yes	No	Yes	No								
0	1	2	1	209	112							0.17	0.95
**Transhumance practiced in the farm**													
Yes	No	Yes	No	Yes	No								
0	1	1	2	48	273							0.67	0.37
**Disinfections performed in the farm**													
Yes	No	Yes	No	Yes	No								
1	0	3	0	293	28							0.76	0.59
**Annual frequency of systemic disinfections in the farm**													
0 – 1 occasion	2 – 10 occasions	> 10 occasions	0 – 1 occasion	2 – 10 occasions	> 10 occasions	0 – 1 occasion	2 – 10 occasions	> 10 occasions					
0	1	0	0	3	0	79	224	18				0.81	0.53
**Daily number of milking sessions**													
1	2	3	1	2	3	1	2	3					
0	1	0	0	3	0	1	260	60				0.89	0.70
**Use of teat disinfection after milking**													
Yes	No	Yes	No	Yes	No								
0	1	0	3	52	269							0.66	0.45
**Grazing practiced**													
Yes	No	Yes	No	Yes	No								
1	0	3	0	279	42							0.70	0.50
**Grazing land available to animals**													
≤ 0.50 ac. per animal	0.51-2.00 ac. per animal	> 2.00 ac. per animal	≤ 0.50 ac. per animal	0.51-2.00 ac. per animal	> 2.00 ac. per animal	≤ 0.50 ac. per animal	0.51-2.00 ac. per animal	> 2.00 ac. per animal					
0	1	0	3	0	0	125	150	46				0.56	0.57
**Annual duration of grazing**													
0 months	1 - 5 months	6 - 10 months	11 – 12 months	0 months	1 - 5 months	6 - 10 months	11 – 12 months	0 months	1 - 5 months	6 - 10 months	11 – 12 months		
0	0	1	0	0	0	2	1	44	46	121	110	0.65	0.69

**Availability of straw bedding**							
Yes	No	Yes	No	Yes	No		
1	0	2	1	265	56	0.64	0.47
**Provision of hay as fodder to animals**							
Yes	No	Yes	No	Yes	No		
1	0	3	0	320	1	0.96	0.92
**Average quantity of hay provided daily to animals during the preceding season**							
≤ 0.6 kg	> 0.6 kg	≤ 0.6 kg	> 0.6 kg	≤ 0.6 kg	> 0.6 kg		
1	0	2	1	106	215	0.16	0.22
**Provision of silage to adult animals**							
Yes	No	Yes	No	Yes	No		
0	1	1	2	71	250	0.59	0.64
**Provision of finished feed (concentrate) to adult animals**							
Yes	No	Yes	No	Yes	No		
1	0	3	0	317	4	0.91	0.85

[1] *p* for associations with isolation of *L. monocytogenes versus* no isolation of this organism.

[2] *p* for associations with isolation of *L. ivanovi versus* no isolation of this organism.

Table S7. Results of univariable analysis for association of human resources-related variables with isolation of *Listeria monocytogenes* or *Listeria ivanovii* from bulk-tank raw milk of 325 sheep farms during a countrywide investigation in Greece.

**Isolation of *L. monocytogens* from the bulk-tank raw milk (*n* = 1)**			**Isolation of *L. ivanovii* from the bulk-tank raw milk (*n* = 3)**			**No isolation of *L. ivanovii* or *L. monocytogens* from the bulk-tank raw milk (*n* = 321)**			***p*** [1]	***p*** [2]
**Age of the farmer**										
Up to 50 years	Over 50 years		Up to 50 years	Over 50 years		Up to 50 years	Over 50 years			
0	1		2	1		195	126		0.21	0.83
**Length of previous farming experience**										
≤ 5 years	> 5 years		≤ 5 years	> 5 years		≤ 5 years	> 5 years			
0	1		2	1		72	249		0.57	0.07
**Education of the farmer**										
Primary	Secondary or post-secondary	Tertiary	Primary	Secondary or post-secondary	Tertiary	Primary	Secondary or post-secondary	Tertiary		
0	1	0	0	2	1	57	222	42	0.80	0.56
**Farmer by profession**										
Yes	No		Yes	No		Yes	No			
1	0		3	0		288	33		0.74	0.56
**Family tradition in farming**										
Yes	No		Yes	No		Yes	No			
1	0		2	1		280	41		0.70	0.30
**Presence of working staff in the flock**										
Yes	No		Yes	No		Yes	No			
1	0		2	1		120	201		0.20	0.30

[1] *p* for associations with isolation of *L. monocytogenes versus* no isolation of this organism.

[2] *p* for associations with isolation of *L. ivanovi versus* no isolation of this organism.

Table S8. Results of univariable analysis for association of climate-related variables with isolation of *Listeria monocytogenes* or *Listeria ivanovii* from bulk-tank raw milk of 325 sheep farms during a countrywide investigation in Greece.

**Isolation of *L. monocytogens* from the bulk-tank raw milk (*n* = 1)**	**Isolation of *L. ivanovii* from the bulk-tank raw milk (*n* = 3)**	**No isolation of *L. ivanovii* or *L. monocytogens* from the bulk-tank raw milk (*n* = 321)**	***p*** [1]	***p*** [2]
	**Average mean temperature at 2 m**			
24.3 °C	14.1 °C ± 6.7 °C	14.1 °C ± 0.4 °C	n/a	0.98
	**Average earth skin temperature**			
25.2 °C	13.8 °C ± 7.3 °C	14.1 °C ± 0.4 °C	n/a	0.93
	**Average minimum temperature at 2 m**			
18.2 °C	9.7 °C ± 6.6 °C	10.2 °C ± 0.4 °C	n/a	0.91
	**Average maximum temperature at 2 m**			
30.1 °C	19.1 °C ± 6.4 °C	18.8 °C ± 0.4 °C	n/a	0.96
	**Average temperature range at 2 m**			
12.07 °C	9.4 °C ± 1.4 °C	8.7 °C ± 0.1 °C	n/a	0.61
	**Average precipitation**			
1.01 mm	0.65 ± 0.35 mm	1.70 ± 0.08 mm	n/a	0.20
	**Average relative humidity at 2 m**			
53.2%	62.2% ± 2.6%	71.9% ± 0.5%	n/a	0.08
	**Average wind speed at 10 m**			
1.4 m s^{-1}	2.2 ± 0.5 m s^{-1}	2.5 ± 0.1 m s^{-1}	n/a	0.68
	**Average all sky insolation incident**			
25.2 Wh m^{-2}	18.4 ± 5.4 Wh m^{-2}	15.8 ± 0.4 Wh m^{-2}	n/a	0.57

[1] *p* for associations with isolation of *L. monocytogenes versus* no isolation of this organism.

[2] *p* for associations with isolation of *L. ivanovi versus* no isolation of this organism.

Table S9. Metrics of the assembled genome of three *L. ivanovii* isolates from bulk-tank raw milk of sheep in Greece.

**Assembly**	**Isolate Reference**		
	NGS01	NGS03	NGS04
# contigs (≥ 0 bp)	22	29	23
# contigs (≥ 1000 bp)	15	16	16
# contigs (≥ 5000 bp)	12	13	12
# contigs (≥ 10000 bp)	11	12	11
# contigs (≥ 25000 bp)	10	11	10
# contigs (≥ 50000 bp)	10	11	10
Total length (≥ 0 bp)	2902994	2907867	2900133
Total length (≥ 1000 bp)	2898774	2899647	2896587
Total length (≥ 5000 bp)	2892401	2893274	2888936
Total length (≥ 10000 bp)	2883946	2884819	2880481
Total length (≥ 25000 bp)	2868943	2869769	2865426
Total length (≥ 50000 bp)	2868943	2869769	2865426
# contigs	20	26	20
Largest contig	783016	782973	782978
Total length	2902270	2906795	2899161
GC (%)	37.02	37.02	37.02
N50	462177	462172	462178
N75	224675	224840	224683
L50	3	3	3
L75	5	5	5
# N's per 100 kbp	0.00	0.00	0.00
Average coverage (%)	75×	80×	105×
CDS	2890	2882	2856
tRNA	61	61	61
tmRNA	1	1	1
rRNA	7	7	7

Table S10. Summary findings of assessment of genomes, by using the tool PHASTER, of three *Listeria ivanovii* isolates obtained from bulk-tank raw milk of sheep in Greece, for the presence of prophages.

**Assembly**	**NGS01**	**NGS03**	**NGS04**
REGION	1	1	1
REGION_LENGTH	27.5Kb	27.5Kb	27.5Kb
COMPLETENESS (score)	incomplete(30)	incomplete(30)	incomplete(30)
SPECIFIC_KEYWORD	portal, integrase	portal, integrase	portal, integrase
REGION_POSITION	NODE_2_length_538063_cov_31.136098:346740-374288	NODE_2_length_538087_cov_32.041520:346745-374294	NODE_2_length_538098_cov_42.442197:346769-374317
TRNA_NUM	0	0	0
TOTAL_PROTEIN_NUM	16	16	16
PHAGE_HIT_PROTEIN_NUM	8	8	8
HYPOTHETICAL_PROTEIN_NUM	8	8	8
PHAGE+HYPO_PROTEIN_PERCENTAGE	100%	100%	100%
BACTERIAL_PROTEIN_NUM	0	0	0
ATT_SITE_SHOWUP	yes	yes	yes
PHAGE_SPECIES_NUM	7	7	7

MOST_COMMON_PHAGE_NAME (hit_genes_count)	PHAGE_Bacill_G_NC_023719(2),PHAGE_Lactob_Iacchus_NC_048084(2),PHAGE_Bacill_SPbeta_NC_001884(2),PHAGE_Lactob_Bromius_NC_048085(2),PHAGE_Lactob_Lpa804_NC_048134(2),PHAGE_Lactob_Bacchae_NC_047924(2),PHAGE_Paenib_PBL1c_NC_048689(1),PHAGE_Paenib_Xenia_NC_028837(1),PHAGE_Paenib_Shelly_NC_041909(1),PHAGE_Bacill_Staley_NC_022767(1),PHAGE_Brevib_Jenst_NC_028805(1),PHAGE_Mycoba_Charlie_NC_023729(1),PHAGE_Lactob_LpeD_NC_048677(1),PHAGE_Lactob_Semele_NC_047926(1),PHAGE_Bacter_Diva_NC_028788(1),PHAGE_Bacill_Mgbh1_NC_041879(1),PHAGE_Bacill_Stills_NC_028777(1),PHAGE_Cronob_ENT39118_NC_019934(1),PHAGE_Bacter_Sitara_NC_028854(1),PHAGE_Salmon_vB_SosS_Oslo_NC_018279(1),PHAGE_Plankt_PaV_LD_NC_016564(1)	PHAGE_Bacill_G_NC_023719(2),PHAGE_Lactob_Bromius_NC_048085(2),PHAGE_Lactob_Bacchae_NC_047924(2),PHAGE_Bacill_SPbeta_NC_001884(2),PHAGE_Lactob_Lpa804_NC_048134(2),PHAGE_Lactob_Iacchus_NC_048084(2),PHAGE_Bacill_Mgbh1_NC_041879(1),PHAGE_Plankt_PaV_LD_NC_016564(1),PHAGE_Brevib_Jenst_NC_028805(1),PHAGE_Paenib_Shelly_NC_041909(1),PHAGE_Bacill_Staley_NC_022767(1),PHAGE_Cronob_ENT39118_NC_019934(1),PHAGE_Bacill_Stills_NC_028777(1),PHAGE_Mycoba_Charlie_NC_023729(1),PHAGE_Paenib_Xenia_NC_028837(1),PHAGE_Lactob_LpeD_NC_048677(1),PHAGE_Bacter_Sitara_NC_028854(1),PHAGE_Lactob_Semele_NC_047926(1),PHAGE_Bacter_Diva_NC_028788(1),PHAGE_Paenib_PBL1c_NC_048689(1),PHAGE_Salmon_vB_SosS_Oslo_NC_018279(1)	PHAGE_Bacill_G_NC_023719(2),PHAGE_Lactob_Iacchus_NC_048084(2),PHAGE_Bacill_SPbeta_NC_001884(2),PHAGE_Lactob_Bacchae_NC_047924(2),PHAGE_Lactob_Bromius_NC_048085(2),PHAGE_Lactob_Lpa804_NC_048134(2),PHAGE_Brevib_Jenst_NC_028805(1),PHAGE_Lactob_Semele_NC_047926(1),PHAGE_Bacter_Sitara_NC_028854(1),PHAGE_Bacill_Staley_NC_022767(1),PHAGE_Lactob_LpeD_NC_048677(1),PHAGE_Paenib_Xenia_NC_028837(1),PHAGE_Bacill_Stills_NC_028777(1),PHAGE_Cronob_ENT39118_NC_019934(1),PHAGE_Paenib_PBL1c_NC_048689(1),PHAGE_Bacter_Diva_NC_028788(1),PHAGE_Mycoba_Charlie_NC_023729(1),PHAGE_Salmon_vB_SosS_Oslo_NC_018279(1),PHAGE_Paenib_Shelly_NC_041909(1),PHAGE_Plankt_PaV_LD_NC_016564(1),PHAGE_Bacill_Mgbh1_NC_041879(1)
FIRST_MOST_COMMON_PHAGE_NUM	2	2	2
FIRST_MOST_COMMON_PHAGE_PERCENTAGE	12.5%	12.5%	12.5%
GC_PERCENTAGE	36.51%	36.51%	36.50%

Table S11. Virulence factor genes detected, by using the tool VirulenceFinder 2.0, in the genome of three *Listeria ivanovii* isolates obtained from bulk-tank raw milk of sheep in Greece.

(a) Isolate NGS01

Data-base	Virulence factor gene	Identity	Query / Template length	Contig	Position in contig	Accession number
listeria	*flaA*	96.06	864 / 864	NODE_1_length_783016_cov_31.348182	264028..264891	NC_003210.1
listeria	*lap*	93.35	2601 / 2601	NODE_9_length_87297_cov_31.925961	17301..19901	NC_003210.1
listeria	*oppA*	94.17	1680 / 1677	NODE_2_length_538063_cov_31.136098	101617..103296	NC_003210.1
listeria	*rli55*	94.04	285 / 283	NODE_1_length_783016_cov_31.348182	739492..739774	NC_003210.1
listeria	*rli60*	90.27	185 / 185	NODE_2_length_538063_cov_31.136098	344374..344557	NC_003210.1
listeria	*rsbv*	90.72	345 / 345	NODE_1_length_783016_cov_31.348182	480151..480495	NC_003210.1
listeria	*clpp*	91.12	597 / 597	NODE_5_length_224675_cov_32.031583	126595..127191	NC_003210.1
listeria	*degU*	90.25	687 / 687	NODE_5_length_224675_cov_32.031583	73998..74684	NC_003210.1
listeria	*fri*	92.08	467 / 471	NODE_1_length_783016_cov_31.348182	530447..530913	NC_003210.1
listeria	*hfq*	93.59	234 / 234	NODE_3_length_462177_cov_28.360333	104637..104870	NC_003210.1
listeria	*inlC*	92.98	869 / 891	NODE_8_length_89443_cov_32.713814	1853..2721	NC_003210.1
listeria	*lhrC*	90.09	111 / 111	NODE_8_length_89443_cov_32.713814	84134..84243	NC_003210.1
listeria	*lhrC*	99.1	111 / 111	NODE_1_length_783016_cov_31.348182	533015..533125	NC_003210.1
listeria	*lsp*	93.76	465 / 465	NODE_2_length_538063_cov_31.136098	488439..488903	NC_003210.1
listeria	*tcsA*	95.62	1074 / 1074	NODE_3_length_462177_cov_28.360333	195054..196127	NC_003210.1
listeria	*tig*	92.76	1284 / 1284	NODE_3_length_462177_cov_28.360333	75600..76883	NC_003210.1

(b) Isolate NGS03

Data-base	Virulence factor gene	Identity	Query / Template length	Contig	Position in contig	Accession number
listeria	*flaA*	96.06	864 / 864	NODE_1_length_782973_cov_32.715747	518128..518991	NC_003210.1
listeria	*lap*	93.35	2601 / 2601	NODE_10_length_87296_cov_32.180947	67396..69996	NC_003210.1
listeria	*oppA*	94.17	1680 / 1677	NODE_2_length_538087_cov_32.041520	101619..103298	NC_003210.1
listeria	*rli55*	94.04	285 / 283	NODE_1_length_782973_cov_32.715747	43240..43522	NC_003210.1
listeria	*rli60*	90.27	185 / 185	NODE_2_length_538087_cov_32.041520	344379..344562	NC_003210.1
listeria	*rsbv*	90.72	345 / 345	NODE_1_length_782973_cov_32.715747	302529..302873	NC_003210.1
listeria	*clpp*	91.12	597 / 597	NODE_5_length_224840_cov_33.193500	126592..127188	NC_003210.1
listeria	*degU*	90.25	687 / 687	NODE_5_length_224840_cov_33.193500	73996..74682	NC_003210.1
listeria	*fri*	92.08	467 / 471	NODE_1_length_782973_cov_32.715747	252099..252565	NC_003210.1
listeria	*hfq*	93.59	234 / 234	NODE_3_length_462172_cov_30.204019	104632..104865	NC_003210.1
listeria	*inlC*	92.87	869 / 891	NODE_5_length_224840_cov_33.193500	1853..2721	NC_003210.1
listeria	*lhrC*	99.1	111 / 111	NODE_1_length_782973_cov_32.715747	249887..249997	NC_003210.1
listeria	*lhrC*	91.96	112 / 111	NODE_9_length_89642_cov_33.152600	5404..5514	NC_003210.1

listeria	*lsp*	93.76	465 / 465	NODE_2_length_538087_cov_32.041520	488450..488914	NC_003210.1
listeria	*tcsA*	95.62	1074 / 1074	NODE_3_length_462172_cov_30.204019	195050..196123	NC_003210.1
listeria	*tig*	92.76	1284 / 1284	NODE_3_length_462172_cov_30.204019	75595..76878	NC_003210.1

(c) Isolate NGS04

**Data-base**	**Virulence factor gene**	**Identity**	**Query / Template length**	**Contig**	**Position in contig**	**Accession number**
listeria	*flaA*	96.06	864 / 864	NODE_1_length_782978_cov_43.621160	518130..518993	NC_003210.1
listeria	*lap*	93.35	2601 / 2601	NODE_9_length_87299_cov_44.829854	17301..19901	NC_003210.1
listeria	*oppA*	94.17	1680 / 1677	NODE_2_length_538098_cov_42.442197	101633..103312	NC_003210.1
listeria	*rli55*	94.04	285 / 283	NODE_1_length_782978_cov_43.621160	43243..43525	NC_003210.1
listeria	*rli60*	90.27	185 / 185	NODE_2_length_538098_cov_42.442197	344403..344586	NC_003210.1
listeria	*rsbv*	90.72	345 / 345	NODE_1_length_782978_cov_43.621160	302524..302868	NC_003210.1
listeria	*clpp*	91.12	597 / 597	NODE_5_length_224683_cov_43.244767	97491..98087	NC_003210.1
listeria	*degU*	90.25	687 / 687	NODE_5_length_224683_cov_43.244767	150001..150687	NC_003210.1
listeria	*fri*	92.08	467 / 471	NODE_1_length_782978_cov_43.621160	252105..252571	NC_003210.1
listeria	*hfq*	93.59	234 / 234	NODE_3_length_462178_cov_42.292641	357310..357543	NC_003210.1
listeria	*inlC*	92.98	869 / 891	NODE_5_length_224683_cov_43.244767	221963..222831	NC_003210.1
listeria	*lhrC*	91.96	112 / 111	NODE_8_length_89649_cov_44.258652	5405..5515	NC_003210.1
listeria	*lhrC*	99.1	111 / 111	NODE_1_length_782978_cov_43.621160	249893..250003	NC_003210.1
listeria	*lsp*	93.76	465 / 465	NODE_2_length_538098_cov_42.442197	488475..488939	NC_003210.1
listeria	*tcsA*	95.62	1074 / 1074	NODE_3_length_462178_cov_42.292641	266050..267123	NC_003210.1
listeria	*tig*	92.76	1284 / 1284	NODE_3_length_462178_cov_42.292641	385297..386580	NC_003210.1

Table S12. Proteins encoded by the virulence factor genes (in alphabetical order) detected in the genome of three *Listeria ivanovii* isolates obtained from bulk-tank raw milk of sheep in Greece.

Virulence factor gene	Protein encoded	Proposed function	Bacterial species in which studied
clpp	ATP-dependent Clp endopeptidase proteolytic subunit ClpP	Peptide cleaver in various proteins in processes requiring ATP hydrolysis; chymotrypsin-like activity; degradation of misfolded proteins	*L. ivanovii*
degU	Two-component system response regulator DegU	DNA binding	*L. ivanovii*
flaA	Flagellin A	Polymerization to forming the filaments of bacterial flagella	*L. ivanovii*
fri	Non-heme iron-binding ferritin Fri	Ferric iron binding; oxidoreductase activity acting on metal ions	*L. ivanovii*
hfq	RNA chaperone Hfq	RNA chaperone binding small regulatory RNA (sRNAs) and mRNAs to facilitate mRNA translational regulation in response to stressing factors; binding with high specificity to tRNAs	*L. ivanovii*
inlC	Internalin C	Virulence enhancer, impairing translocation of host transcription factor NF-kappa-B to the nucleus and antagonizing the function of Tuba dynamin-binding protein, thus promoting bacterial spreading; perturbing morphology of host cell junctions by impairing host DNMBP (Tuba) and WASL interaction, altering cortical tension at the cell junctions and allowing bacteria to more efficiently form bacteria-filled cell protrusions, thus promoting bacterial spreading within infected tissues; down-regulator of host inflammation response; interaction with host I-kappa-B kinase alpha (IKKA, CHUK), preventing IKKA from phosphorylating NF-kappa-B inhibitor alpha (IKBA, NFKBIA) and thus delaying degradation of phospho-IKBA; impairing translocation of host transcription factor p65 (a subunit of NF-kappa-B, RELA) into the nucleus, thus preventing activation of NF-KB-regulated genes	*L. monocytogenes*
lap	Adhesion-mediating acetaldehyde/alcohol dehydrogenase LAP	Acetaldehyde dehydrogenase (acetylating) activity	*L. monocytogenes*
lsp	Lipoprotein signal peptidase	Catalyzing removal of signal peptides from prolipoproteins	*L. ivanovii*
oppA	Putative oligopeptide ABC transporter-binding protein	Binding protein; ATP-binding cassette (ABC) transporter complex, transmembrane transport	*L. ivanovii*
rsbv	Anti-sigma b factor antagonist RsbV	Anti-sigma factor antagonist activity	*L. monocytogenes*
tcsA	CD4+ T cell-stimulating antigen, lipoprotein	Lipoprotein, putative CD4+ T cell-stimulating antigen	*L. ivanovii*
tig	Trigger factor	Protein export; chaperone protein by maintaining newly synthesized proteins in open conformation; peptidyl-prolyl *cis-trans* isomerase	*L. ivanovii*

Table S13. The output of CRISPRCasFinder for the genome of three *Listeria ivanovii* isolates obtained from bulk-tank raw milk of sheep in Greece.

(a) Isolate NGS01

Element	CRISPR Id / Cas Type	Start	End	Spacer / Gene	Repeat consensus / cas genes	Direction
CRISPR	NODE_1_length_783016_cov_31_348182_1	36936	37222	4	GTTTTAATTACTTATTGTGAAATGTAAAT	ND [1]
Cas cluster	General-Class1	47089	55206	8	Cas6_0_I-III, Cas8a1_0_IB, Cas7_1_IB, Cas5_0_IB, Cas3_0_I, Cas4_0_I-II, Cas1_0_I-II-III, Cas2_0_I-II-III-V	
CRISPR	NODE_1_length_783016_cov_31_348182_2	55405	55887	7	GTTTTAATTACTTATTATGAAATGTAAAT	-
CRISPR	NODE_1_length_783016_cov_31_348182_3	325941	326341	6	ACAACCAGCTAACGAATTTAGAT	+
CRISPR	NODE_4_length_347955_cov_33_429845_1	145397	145509	1	AACGCGAGGATTTAATCCTTCGTGGAAGTTTCAAAA	+

(b) Isolate NGS03

Element	CRISPR Id / Cas Type	Start	End	Spacer / Gene	Repeat consensus / cas genes	Direction
CRISPR	NODE_1_length_782973_cov_32_715747_1	456678	457078	6	ATCCAAATTCGTTAGCTGGTTGT	ND
CRISPR	NODE_1_length_782973_cov_32_715747_2	727086	727568	7	ATTTACATTTCATAATAAGTAATTAAAAC	ND
Cas cluster	General-Class1	727767	735884	8	Cas2_0_I-II-III-V, Cas1_0_I-II-III, Cas4_0_I-II, Cas3_0_I, Cas5_0_IB, Cas7_1_IB, Cas8a1_0_IB, Cas6_0_I-III	
CRISPR	NODE_1_length_782973_cov_32_715747_3	745753	746039	4	ATTTACATTTCATAATAAGTAATTAAAAC	ND
CRISPR	NODE_4_length_238231_cov_34_868230_1	35676	35788	1	AACGCGAGGATTTAATCCTTCGTGGAAGTTTCAAAA	+

(c) Isolate NGS04

Element	CRISPR Id / Cas Type	Start	End	Spacer / Gene	Repeat consensus / cas genes	Direction
CRISPR	NODE_1_length_782978_cov_43_621160_1	456677	457077	6	ATCCAAATTCGTTAGCTGGTTGT	ND
CRISPR	NODE_1_length_782978_cov_43_621160_2	727090	727572	7	ATTTACATTTCATAATAAGTAATTAAAAC	ND
Cas cluster	General-Class1	727771	735888	8	Cas2_0_I-II-III-V, Cas1_0_I-II-III, Cas4_0_I-II, Cas3_0_I, Cas5_0_IB, Cas7_1_IB, Cas8a1_0_IB, Cas6_0_I-III	
CRISPR	NODE_1_length_782978_cov_43_621160_3	745756	746042	4	ATTTACATTTCATAATAAGTAATTAAAAC	ND

CRISPR	NODE_3_length_462178_cov_42_292641_1	432571	432661	1	AAATTTTTTCCAGTTTGTTTAAATT	ND
CRISPR	NODE_4_length_347079_cov_44_764858_1	144514	144626	1	AACGCGAGGATTTAATCCTTCGTGGAAGTT TCAAAA	+

[1] Not Defined.
